# Coexistence of anhedonia and sensation-seeking with context-dependent gut dysbiosis in a prodromal transgenic mouse model of Parkinson’s disease

**DOI:** 10.1038/s41398-026-04306-w

**Published:** 2026-07-27

**Authors:** Olga Dubljević, Dušanka Popović, Milica Potrebić Stefanović, Branka Petković, Emilija Brdarić, Franziska Richter, Vesna Pešić

**Affiliations:** 1 Laboratory of Molecular Neurobiology and Behavior, Department of Neurobiology, Institute for Biological Research “Siniša Stanković” – National Institute of the Republic of Serbia, University of Belgrade, Bulevar despota Stefana 142, 11108 Belgrade, Serbia; 2 Immunotoxicology Group, Department of Ecology, Institute for Biological Research “Siniša Stanković” – National Institute of the Republic of Serbia, University of Belgrade, Bulevar despota Stefana 142, 11108 Belgrade, Serbia; 3 Department of Neurophysiology, Institute for Biological Research “Siniša Stanković” - National Institute of the Republic of Serbia, University of Belgrade, Bulevar despota Stefana 142, 11108 Belgrade, Serbia; 4 Group for Probiotics and Microbiota-Host Interaction, Department of Microbiology and Plant Biology, Institute of Molecular Genetics and Genetic Engineering, University of Belgrade, Vojvode Stepe 444a, 11042 Belgrade, Serbia; 5 Department of Pharmacology, Toxicology and Pharmacy, University of Veterinary Medicine Hannover, Hannover, Germany; 6 Center for Systems Neuroscience (ZSN) Hannover, Hannover, Germany

**Keywords:** Neuroscience, Diseases

## Abstract

Accumulating evidence indicates that gut microbiota plays a role in the progression of Parkinson’s disease (PD). Microbiota-based treatments for prodromal PD, which is accompanied by a wide range of neuropsychiatric symptoms, are being considered a potential early intervention but are still under-investigated. This study examined the association between symptoms of affective and motivational impairments and gut dysbiosis in a transgenic mouse model (line G2-3) that overexpresses A53T mutant α-synuclein, a key protein involved in the disease pathology. By introducing biologically meaningful variations, such as genotype-based social grouping, efforts have been made to strengthen validity of the results and their translational relevance. Behavioral assessment was conducted on 6-month-old mice (the premotor stage) using Open Field, Elevated Plus Maze, Sucrose Preference and Tail Suspension tests. Fecal microbiota of mice was analyzed using 16S rRNA gene sequencing. Obtained data revealed a strong behavioral phenotype in transgenic mice expressed as co-occurrence of consummatory anhedonia and sensation-seeking/risk-taking behavior, without significant motivational deficits and with variable gut dysbiosis: mixed-genotype housing, among others, prominently favored Bacteroidaceae/*Bacteroides*, as observed in PD patients, while single-genotype housing increased *Dubosiella*, a key bacteria in gut-originated PD model, along with the enrichment of *Bifidobacterium pseudolongum*, a well-known health-promoting bacterium. Overall, using a mouse model with adequate construct validity, this study identifies challenges in novel strategies that consider gut microbiota as an early diagnostic and therapeutic target for PD; it highlights the potential of the examined transgenic strain in studying low hedonic tone- and sensation-seeking-associated PD prodrome, building the basis for personalized medicine.

## Introduction

Parkinson’s disease (PD) is a progressive neurodegenerative disorder diagnosed based on characteristic motor symptoms. It is often preceded and accompanied by a wide range of neuropsychiatric symptoms and is increasingly recognized as a neuropsychiatric disorder [1, 2]. At present, no therapy can slow down or stop the progression of PD [3]. Identification of prodromal symptoms provides a therapeutic window for therapies that modify the course of the disease, and optimal treatment is recognized in a multidisciplinary approach and the inclusion of non-pharmacological interventions [3, 4].

Accumulating evidence indicates that gut microbiota plays a role in the disease progression [5, 6] and personalized microbiota-based treatments for prodromal PD are considered as potential early intervention [7]. Therefore, comprehensive animal models that yield robust findings on the association between the microbiota and PD-related early behavioral symptomatology are of utmost importance, but studies are limited. Alpha-synuclein (α-synuclein) related pathology is at the core of the pathogenetic processes seen in PD and mouse models that overexpress human wild-type or different *SNCA* mutations have high construct validity for preclinical research [8].

In the present study we aimed to assess the association between gut dysbiosis and the neuropsychiatric symptoms in a transgenic mouse model (line G2-3) that is constructed to overexpress A53T mutant human α-synuclein, a key protein involved in the disease pathology [9]. The A53T mice have been recognized as a promising preclinical model of prodromal synucleinopathies as they exhibit constipation and olfactory dysfunction, well-known prodromal symptoms of the PD, before the onset of motor dysfunction [10, 11]. Literature suggests a dynamic progression in the spreading and/or accumulation of toxic α-synuclein in the enteric nervous system of presymptomatic mice making the G2–3 line a unique tool for examining the gut–brain connection in the progression of PD [10]. Moreover, our previous findings have indicated (for the first time) that the neurobehavioral phenotype of this mouse line in the premotor phase contains elements associated with ADHD [12], which is of clinical relevance [13].

The focus was on examining anxiety-like, depression-like, anhedonia-like and motivated behaviors, as disturbed affect and motivation are major prodromal and non-motor neuropsychiatric manifestations of the PD [1, 2, 14]. These disturbances are described in adult ADHD as well and are associated with microbiota independently of PD [15, 16]. Considering the requirement that the validity of preclinical animal studies needs to be improved to ensure their translational success, efforts have been made to strengthen internal and external validity of the study outcomes [17–19]. Because mice are coprophagic, social grouping of disease-prone and healthy mice may influence the composition of their microbiota, blurring the influence of genotype/disease. Also, different social microenvironments may pose varying challenges to animals of different genotypes due to their peculiarities in psychological perception of and biological response to social demands [20]. Accordingly, the potentially confounding effects related to control group selection [21, 22] and genotype-based social grouping [23, 24] have been addressed. Our emphasis on establishing face validity of the prodrome in the model stems from the fact that neuropsychiatric disorders are primarily diagnosed based on behavioral, emotional and cognitive features [25].

Answering a simple biological question—whether the expression of a pathological form of alpha-synuclein, implicated in the pathogenesis of the PD, has robust behavioral and gut microbiota phenotypes before the motor symptoms onset—helps to identify challenges in novel strategies seeing the gut microbiota as early diagnostic and therapeutic target for PD [7, 26]. Moreover, defining a robust prodromal behavioral phenotypes in transgenic mouse models of PD should be viewed as an imperative per se in translational research, since distinct prodromal subtypes of the PD are clinically recognized as well as the need to understand the neuropsychiatric manifestations of the disease and predict individual responses to specific therapies [1, 27, 28].

## Material and methods

### Animals

Hemizygous A53T male carriers (line G2-3; JAX006823, https://www.jax.org/strain/006823; congenic C57BL/6J) were purchased from the Jackson Laboratory. To maintain a colony, they were bred to C57BL/6J females, belonging to the C57BL/6J breeders previously arrived from the Charles River (Germany). Mice were housed in an institutional vivarium, under standard housing conditions and under the control of a veterinarian. Once visually pregnant, females were removed from the harem cage to a separate cage with nesting material and housed singly until the litter was weaned. The genotype of the offspring (A53T carriers, Tg; A53T non-carriers, NonTg) was revealed by PCR analysis of tail DNA sampled at the time of weanling (postnatal day 23 ± 2). Second generation offspring were used in the experiments. In accordance with the study’s goals, and considering that both NonTg and C57BL/6J mice were suggested as controls for this strain (https://www.jax.org/strain/006823, last accessed on 21 July 2026), males of the C57BL/6J strain were included as well.

All animal procedures followed EU Directive 2010/63/EU and were approved by the Ethical Committee of the Institute and by the National Ethic Research Committee (323–07-02149/2023–05).

### Experimental procedure

Depending on the group assignment, male mice of different genotypes (Tg, NonTg) were either co-housed within a cage (mixed-genotype housing (MGH), balanced by genotype) or separated by the genotype (single-genotype housing, SGH). Groups were formed with P29 ± 1 day old mice considering that, in addition to the first weeks of life, a period between weaning and young adulthood is another window of opportunity to influence microbiome composition [29]. A home-cage group composition (4–6 mice per cage) was not changed until the end of the experiment. C57BL/6J mice were housed under the same conditions and were not co-housed with mice of the JAX006823 strain.

The experiment was performed on 6-months-old male mice, belonging to 5 groups (*n* = 12 mice per group): C57BL/6J, TgMGH, NonTgMGH, TgSGH, NonTgSGH. All mice were exposed to a battery of four behavioral tests, day-by-day and always in the same order: 1) the open field test (OFT), to confirm previously reported novelty-induced hyperactivity of the strain [12, 30, 31], an indication of sensation-seeking as a trait [32, 33]; 2) elevated plus maze test (EPM), to assess anxiety-like and sensation-seeking/risk-taking behavior, considering that the open arms of the EPM are a source of the anxiogenic stimulus [34]; rodents typically avoid open arms of the EPM [35] and their preference implies tendencies toward risk-taking within the framework of sensation-seeking ([36]; for behavioral and biological correlates of sensation-seeking please see [37]); 3) tail suspension test (TST), to assess depression-like behavior, through decreased active coping with an imposed stressful situation [36]; 4) sucrose preference test (SPT), to assess consummatory aspect of hedonic response [38, 39], along with food intake as an apathy-related behavior [40]; food consumption as a goal-directed and motivated behavior indispensable for survival, and reduced motivation to eat was used as a symptom of apathetic behavior that does not affect a preference for the sweet [41].

Fecal sampling (*n* = 8 mice per group; randomly selected) was performed before testing (schematic presentation of the experimental design is shown in Supplementary Fig. 1). One mouse was excluded from the NonTgSGH group due to a corrupted video recording of behavior in the EPM test, so the number of mice in this group for this test was reduced to 11.

For a detailed description of the methodological procedure, see Supplementary Material.

### Statistical analysis

The data were statistically analyzed using Statistica 12 software (StatSoft Inc., Tulsa, OK, United States). The normality of data sets was estimated by Shapiro-Wilk’s test. Datasets for locomotor and vertical activity and latency to the first immobility were transformed (sqrt) to achieve normality.

To estimate the influence of genotype and genotype-based social grouping, a two-way analysis of variance (ANOVA) with Tukey HSD post hoc (unequal N HSD in the case of EPM data) was used whenever normality of data sets was satisfied. Planned comparisons were performed even in the absence of significant interactions as comparisons among treatment means remain valid even when factors do not interact [42]. For non-parametric data, Mann Whitney *U*-test was used. Comparison with the C57BL/6J group was performed using *t*-test or *U*-test, depending on normality distribution. The group preference for open arms in the EPM and sucrose solution in the SPT was calculated using one-sample *t*-test.

The *U*-test was used to detect differentially abundant microbiota at phylum, class, order, family and genus (relative abundance > 0.01) levels. Linear discriminant analysis (LDA) of effect size (LEfSe) was used to examine the abundance of dominant species, with the threshold for logarithmic LDA score set at 2.0. The accepted level of significance was *p* ≤ 0.05 (two-tailed).

## Results

### Behavior of mice in the open field, sucrose preference test and tail suspension test

#### Open field test

**Fig. 1:**
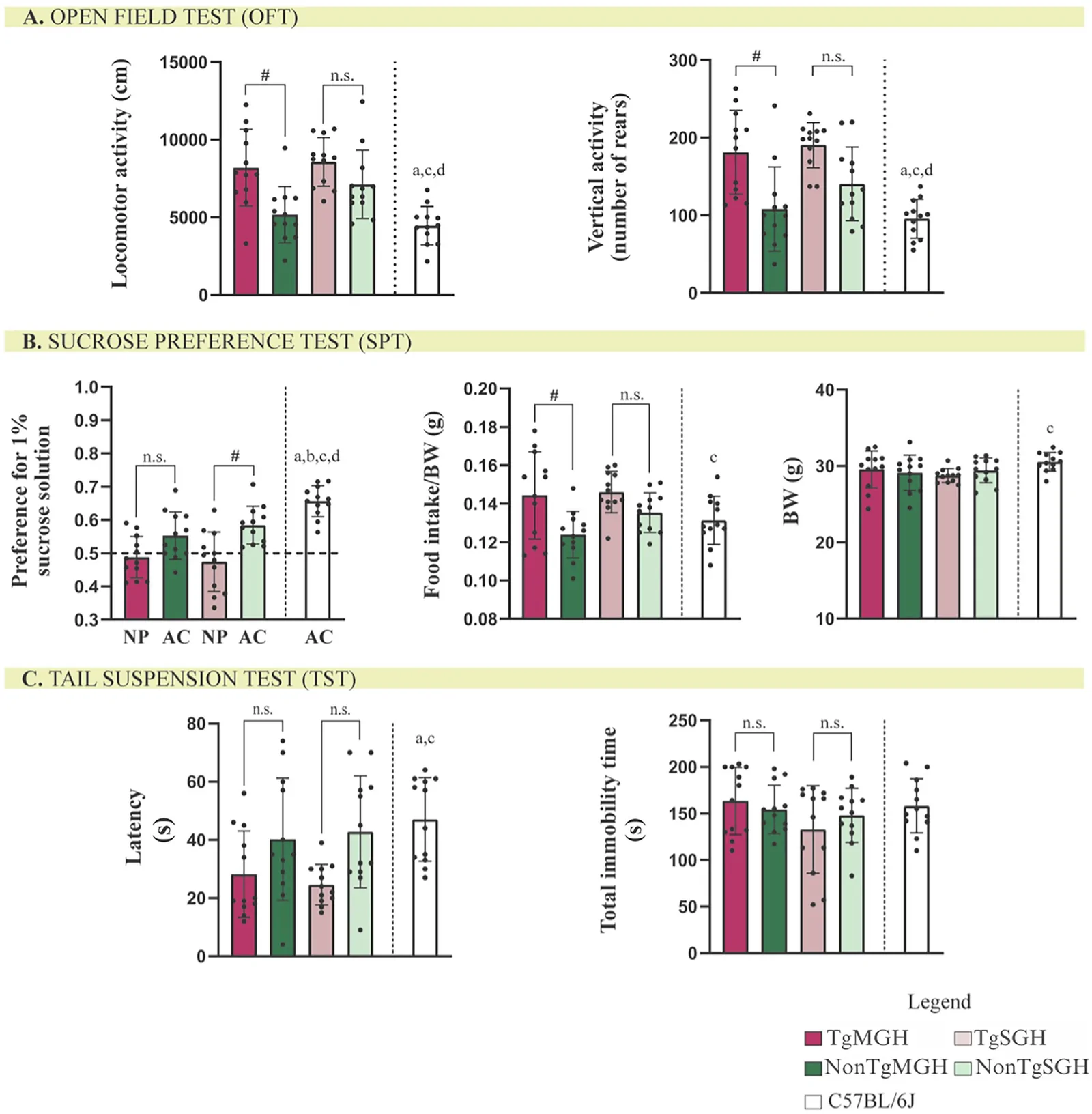
Behavioral parameters of transgenic and non-transgenic mice of the JAX006823 strain and C57BL/6J mice in the open field, sucrose preference, and tail suspension test. **A** Locomotor activity (cm) and vertical activity (number of rears) of mice in the open field test. **B** The preference for 1% sucrose solution, food intake (g) per body weight (BW, g), and BW. **C** The latency to the first immobility (s) and total immobility time (s) of mice in the tail suspension test. Results are presented as mean ± SD. Tg – transgenic, NonTg – non-transgenic, MGH – mixed-genotype housing, SGH – single-genotype housing. *n* = 12 per group; ^#^*p* < 0.05, Tukey HSD test; ^a^*p* < 0.05 *vs*. TgMGH, ^b^*p* < 0.05 *vs*. NonTgMGH, ^c^*p* < 0.05 *vs*. TgSGH, ^d^*p* < 0.05 *vs*. NonTgSGH, *t*- or *U*-test. NP – no preference, AC – above the chance level (0.5), one sample *t*-test.

Two-way ANOVA revealed significant influence of genotype and genotype-based social grouping on locomotor activity (F_(1,44)_ = 15.209, *p* < 0.001 and (F_(1,44)_ = 4.874, *p* = 0.032, respectively) and genotype on vertical activity (F_(1,44)_ = 20.282, *p* < 0.001) of JAX006823 littermates. Post-hoc comparisons showed hyperactivity in TgMGH compared to NonTgMGH mice for both locomotor and vertical activity (Fig. 1A; ^#^*p* = 0.003, Tukey HSD), which was not the case with SGH groups. C57BL/6J group was less active than TgMGH, TgSGH and NonTgSGH groups regarding both locomotor and vertical activity (Fig. 1A; ^a,c,d^*p* < 0.01 for all comparisons, t-test).

#### Sucrose preference test

Genotype emerged as a factor that influences sucrose preference in JAX006823 littermates (F_(1,44)_ = 18.096, *p* < 0.001); post-hoc comparisons revealed no difference between TgMGH and NonTgMGH mice and significantly increased preference of NonTgSGH compared to TgSGH mice (Fig. 1B; ^#^*p* = 0.003, Tukey HSD). However, one-sample *t*-test revealed no preference in Tg mice regardless of housing conditions (*p* > 0.339), defining them as anhedonic per se. Importantly, C57BL/6J mice showed higher preference for sucrose solution compared to all congenic JAX006823 groups (Fig. 1B; ^a,b,c,d^*p* < 0.003, *t*-test). Interestingly, in MGH conditions Tg mice consumed more food per gram of body weight (BW) than NonTg mice (Fig. 1B; ^#^*p* = 0.008, Tukey HSD), without significant differences in BW (total caloric intake is given as supplementary data); there were no significant differences between C57BL/6J mice and congenic JAX006823 strain except with TgSGH group compared to which they ate less and weighed more.

#### Tail suspension test

The latency to the first immobility was influenced by genotype (F_(1,44)_ = 8.338, *p* = 0.006) but post-hoc comparisons did not reveal significant between-group differences (Fig. 1C). The latency observed in C57BL/6J mice was higher than in TgMGH and TgSGH groups (Fig. 1C, ^a,c,^*p* < 0.01, *U*-test). There was no difference in total immobility time between experimental groups.

### Behavior of mice in the elevated plus maze test

#### Open arms (OA)

**Fig. 2:**
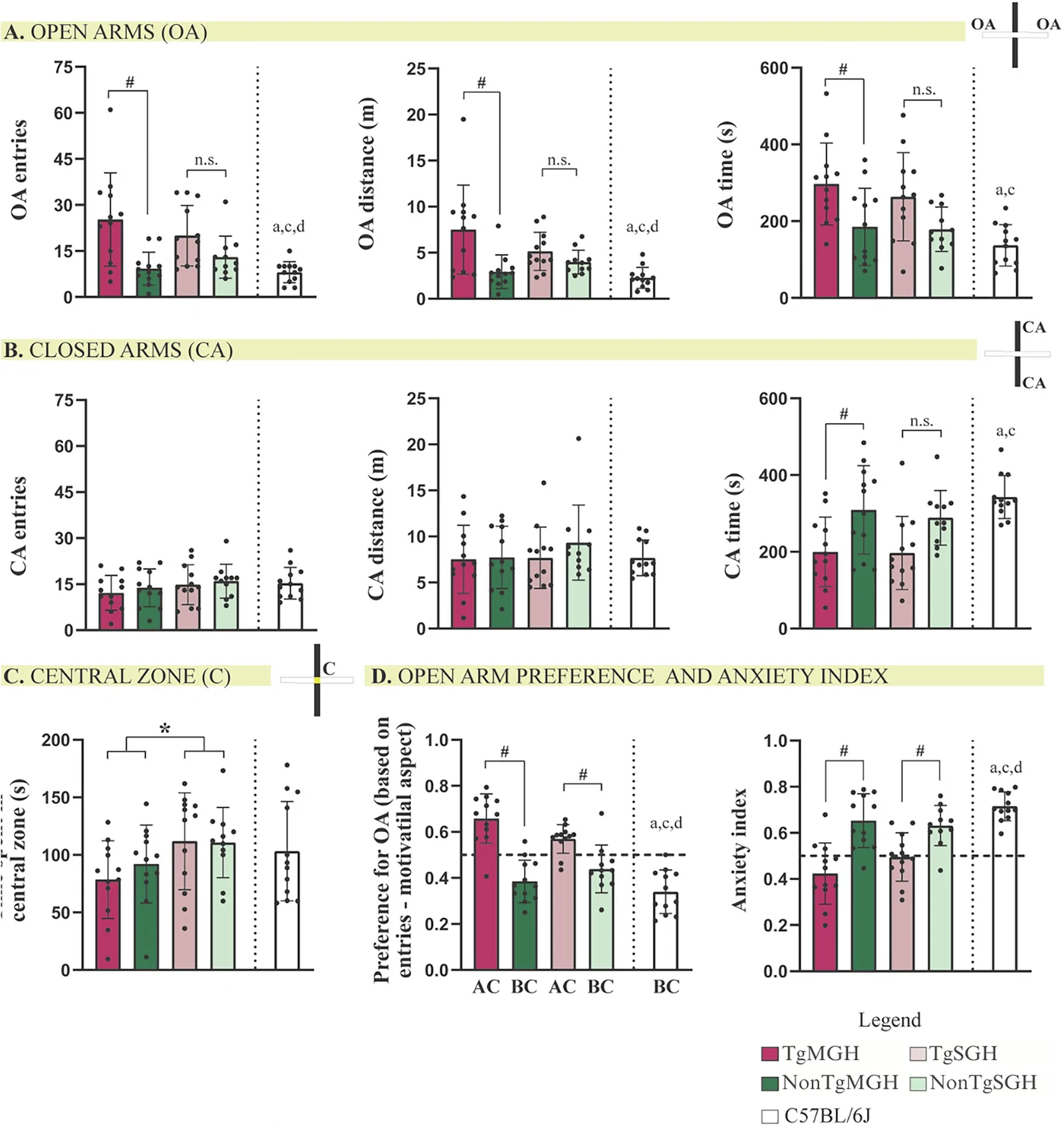
Behavioral parameters of transgenic and non-transgenic mice of the JAX006823 strain and C57BL/6J mice in the elevated plus maze test. **A** Behavior related to the open arms: number of entries, distance (m), and time spent (s). **B** Behavior related to the closed arms: number of entries, distance (m), and time spent (s). **C** Time spent in the central zone (s). **D** Open arms preference (calculated as number of open arm entries / (number of open + closed arm entries) and anxiety index (calculated as 1 − [([Open arms time/Test duration] + [Open arms entries/Total number of entries])/2], for the details please see Supplementary material). Results are presented as mean ± SD. Tg – transgenic, NonTg – non-transgenic, MGH – mixed-genotype housing, SGH – single-genotype housing. *n* = 12 per group except for NonTgSGH where *n* = 11; AC – above the chance level (0.5), BC – below the chance level, one sample *t*-test. ^#^*p* < 0.05, Unequal N HSD; *p < 0.05, significant influence of genotype-based social grouping; ^a^*p* < 0.05 *vs*. TgMGH, ^c^*p* < 0.05 *vs*. TgSGH, ^d^*p* < 0.05 *vs*. NonTgSGH, *t*-test.

For all examined parameters, genotype emerged as a factor with significant influence (Fig. 2A; number of entries (F_(1,43)_ = 16.740, *p* < 0.001), distance travelled (F_(1,43)_ = 13.342, *p* < 0.001), and time spent (F_(1,43)_ = 11.765, *p* = 0.001). Unequal N HSD revealed that TgMGH compared to NonTgMGH mice had more OA entries (^#^*p* = 0.001), more distance travelled (^#^*p* = 0.001) in and more time spent (^#^*p* = 0.038) in OA. These differences were not evident for SGH groups. C57BL/6J mice showed less OA-related activity than TgMGH, TgSGH and NonTgSGH groups (Fig. 2A; ^a,c,d^*p* < 0.03, *t*-test).

#### Closed arms (CA)

There was no significant difference in CA entries and distance travelled between experimental groups (Fig. 2B). For time spent in CA, significant effect of genotype was observed (F_(1,43)_ = 13.197, *p* < 0.001), while post-hoc analysis revealed that TgMGH mice spent less time in CA than NonTgMGH mice (Fig. 2B; ^#^*p* = 0.035), which was not observed in mice grown in SGH conditions. CA-related activity of C57BL/6J mice differed from the behavior of other experimental groups only regarding CA time, being higher than in TgMGH and TgSGH groups (Fig. 2B; ^a,c^*p* < 0.001, *t*-test).

#### Time spent in the central zone

This emerged as the only parameter influenced solely by genotype-based social grouping (Fig. 2C; F_(1,43)_ = 6.336, *p* = 0.016), by means that MGH groups spent less time in the central zone than SGH groups. The score of C57Bl/6J group did not differ compared to other groups.

#### Open arms preference and anxiety index

Deliberate analysis was performed to estimate OA preference (Fig. 2D), as rodents generally avoid OA of the EPM. Based on number of entries, as a motivational indicator, one-sample *t*-test revealed that OA preference is significantly above the chance level (0.5) in TgMGH and TgSGH groups (^AC^*p*<0.001 and ^AC^*p* = 0.002, respectively) and significantly below in NonTgMGH (^BC^*p*=0.001), NonTgSGH (^BC^*p*=0.039) and C57BL/6J group (^BC^*p*<0.001). Open arms preference was higher in Tg compared to NonTg groups in both MGH and SGH conditions (^#^*p* < 0.001 and ^#^*p* = 0.003, respectively, *U*-test). Anxiety index was influenced by genotype (F_(1,43)_ = 31.121, *p* < 0.001), and post-hoc analysis revealed that it is significantly lower in Tg compared to NonTg groups in both MGH and SGH conditions (Fig. 2D; ^#^*p* < 0.001 and ^#^*p* = 0.033, respectively, Unequal N HSD). In C57BL/6J group it was higher than in all other groups except NonTgMGH (Fig. 2D; ^a,c,d^*p* ≤ 0.02, *t*-test).

### Venn diagram and alpha diversity

**Fig. 3:**
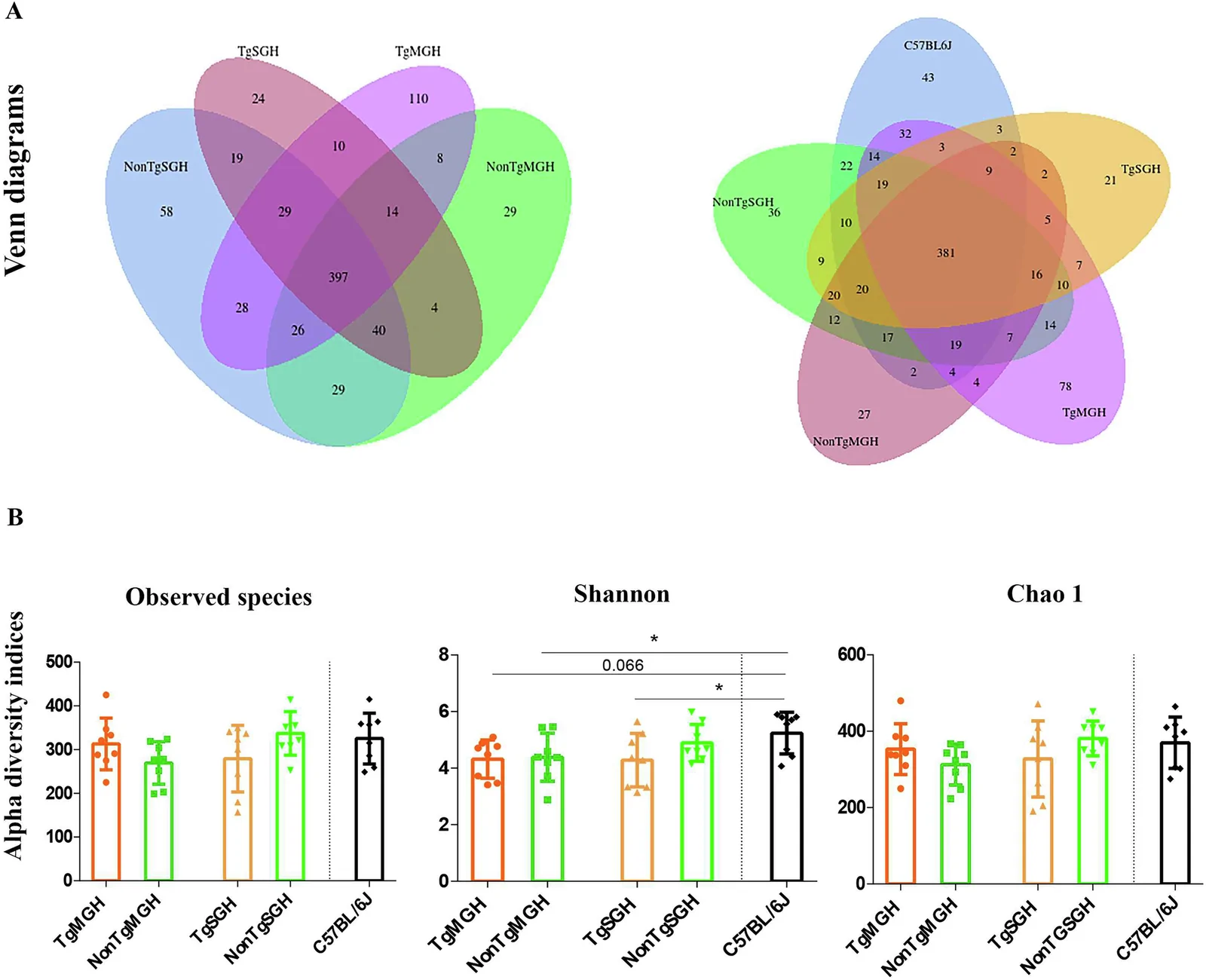
The Venn diagram and alpha diversity analysis of fecal microbiota of transgenic and non-transgenic mice of the JAX006823 strain and C57BL/6J mice. **A, left panel** Bacterial community in four congenic groups of the JAX006823 strain (TgMGH, NonTgMGH, TgSGH, NonTgSGH) contained 397 shared OTUs, which is 48% of all OTUs detected for these groups; the number of unique/group-specific OTUs was the highest in TgMGH (110) and the lowest in TgSGH (24). **A, right panel** Comparison of shared taxa among C57BL/6J and the 4 groups of congenic JAX006823 strain revealed 381 OTUs, which is 43.9% of all detected taxa; in this combined analysis the number of unique OTUs in Tg and NonTg mice decreased, the most in the TgMGH group (by 32, to 78) and the NonTgSGH group (by 22, to 36) and the least in the TgSGH group (by 3, to 21) and the NonTgMGH group (by 2, to 27), indicating that some OTUs that are unique for the littermates of congenic JAX006823 strain are common for C57BL/6J mice and represent approaching the healthy microbiota composition. **B** Alpha diversity metrics based on the Observed species, Shannon index (*0.031≤*p* ≤ 0.041, *U*-test) and Chao1 index; *n* = 8 per group; Tg – transgenic, NonTg – non-transgenic, MGH – mixed-genotype housing, SGH – single-genotype housing.

According to the annotated operational taxonomic units (OTUs), flower diagram was generated (Fig. 3). For the congenic groups of the JAX006823 strain (Fig. 3A, left panel) a common core of bacterial community was represented through shared 397 OTUs, which represents 48% of OTUs detected for these groups. Comparison of shared taxa among C57BL/6J and the 4 groups of congenic JAX006823 strain (Fig. 3A, right panel) revealed shared 381 out of 868 OTUs, which represents 43.9% of all detected taxa. Additional details are provided in the figure legend.

In order to examine gut microbiota community within defined groups, we performed alpha diversity metrics (Fig. 3B). There was no significant influence of genotype and genotype-based social grouping on Observed species, Shannon index (an estimator of specie evenness – relative abundance of each species), and Chao1 index (representing microbial richness), as indicated by two-way ANOVA. However, according to Shannon index, significant decrease in microbiota diversity was noted in TgSGH and NonTgMGH groups compared to C57BL/6J group (*p < 0.041, *U*-test), while TgMGH showed borderline significance (*p* = 0.066, *U*-test).

### Beta diversity

**Fig. 4:**
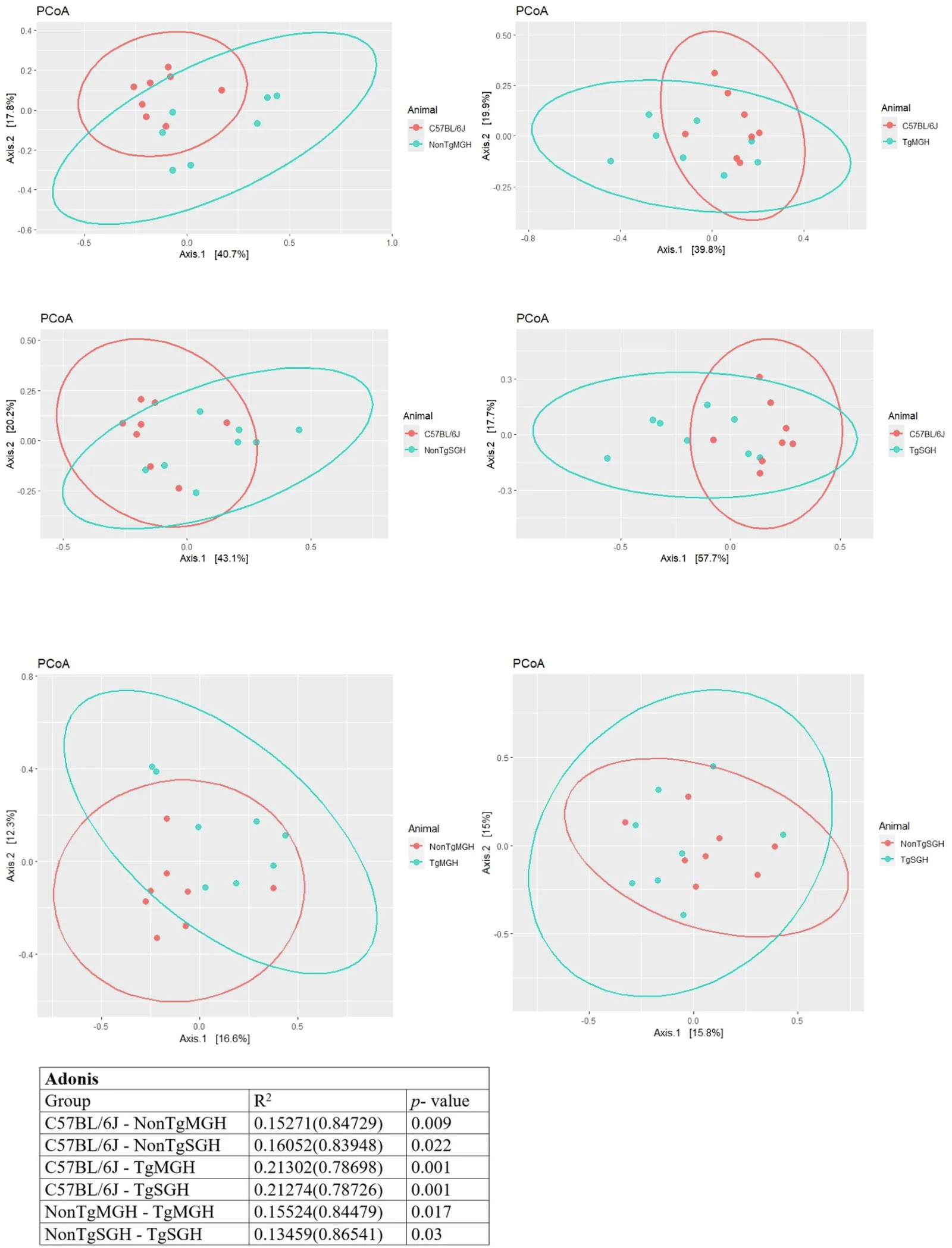
The beta diversity analysis of fecal microbiota of transgenic and non-transgenic mice of the JAX006823 strain and C57BL/6J mice. PCoA, conducted at the genus level, revealed that differences in gut bacterial community exist in all congenic groups of the JAX006823 strain (TgMGH, NonTgMGH, TgSGH, NonTgSGH) compared to C57BL/6J mice as well as between the Tg and NonTg groups in both genotype-based social grouping (MGH and SGH); *n* = 8 per group. The Adonis test, the results of which are shown in the table given within the figure, revealed significant differences between all comparisons of interest. Tg – transgenic, NonTg – non-transgenic mice.

Comparative analysis of microbial community was performed through Principal coordinates analysis (PCoA) based on the genus level (Fig. 4). In PCoA plot based on the Bray Curtis distance matrix it was obvious that differences in gut bacterial community were present between all four congenic groups and C57BL/6J group and between the Tg and NonTg groups in MGH and SGH conditions.

To evaluate inter-group community structure variability, the Adonis test was performed (table within Fig. 4), revealing significant differences between all comparisons of interest. It could be seen that 15.5% of variability was responsible for differences between NonTgMGH and TgMGH groups, 13.5% between NonTgSGH and TgSGH, about 15% between C57BL/6J and NonTg groups and about 21% between C57Bl/6J and Tg groups.

### Analysis at different taxonomic levels, based on the 13 most dominant genera

Taxonomic analysis at phylum classification level revealed dominance of Bacteroidota and Firmicutes followed by Actinobacteriota and Proteobacteria, representing more than 90% of total bacterial count in each group (Supplementary Fig. 2A). At the ordo level, the most dominant were Bacteroidales, Lactobacillales, Bifidobacteriales, Lachnospirales, Erysipelotrichales, Coriobacteriia, Oscillospirales, and Enterobacterales (Supplementary Fig. 2C). The most dominant families were Prevotellaceae, Lactobacillaceae, Bifidobacteriaceae, Lachnospiraceae, Muribaculaceae, Erysipelotrichaceae, Eggerthellaceae, Bacteroidaceae and Oscillospiraceae (Supplementary Fig. 2D). As the most dominant genera emerged *Ligilactobacillus*, *Bifidobacterium, Lactobacillus, Allobaculum, Dubosiella, Enterorhabdus, Lachnospiraceae NK4A136, Limosilactobacillus, Ileibacterium, Roseburia, Bacteroides*, *A2*, and *Escherichia-Shigella* (Supplementary Fig. 2E).

Detailed statistical analysis was performed, by comparing relative abundance of all mentioned taxa between experimental groups, and obtained findings were presented in Table 1.

**Table 1:** The statistical summary of fecal microbiota abundance in transgenic (Tg) and non-transgenic (NonTg) mice of the JAX006823 strain and C57BL/6J mice, at different dominant taxa; *n* = 8 per group.

Genotype-based housing	Mixed (MGH)		Single (SGH)		MGH	SGH
Genotype	Tg	NonTg	Tg	NonTg	Tg	Tg
compared to (*vs*.)	C57BL/6J		C57BL/6J		NonTg	NonTg
Test: Mann-Whitney *U*-test	*U*, *p*	*U*, *p*	*U*, *p*	*U*, *p*	*U*, *p*	*U*, *p*
p: **Bacteroidota**	25, 0.495	**6, 0.007**↓	18, 0.156	**8, 0.014**↓	20, 0.227	27, 0.637
**c: Bacteroidia**	25, 0.495	**6, 0.007**↓	18, 0.156	**8, 0.014**↓	20, 0.227	27, 0.637
o: Bacteroidales	25, 0.495	**6, 0.007**↓	18, 0.156	**8, 0.014**↓	20, 0.227	27, 0.637
f: Prevotellaceae	21, 0.270	16, 0.104	26, 0.564	14, 0.066	**10, 0.024**↑	17, 0.128
f: Muribaculaceae	**3, 0.003**↓	18, 0.156	**12, 0.041**↓	19, 0.189	14, 0.066	27, 0.637
f: Bacteroidaceae	**5, 0.005**↑	32, 0.958	29, 0.793	32, 0.958	**5, 0.005**↑	31, 0.916
**g:** ***Bacteroides***	**5, 0.005**↑	32, 0.958	29, 0.793	32, 0.958	**5, 0.005**↑	31, 0.916
**Firmicutes/ Bacteroidota ratio**	25, 0.495	**5, 0.005**↑	26, 0.564	**12, 0.041**↑	19, 0.189	27, 0.637
p: **Firmicutes**	26, 0.564	17, 0.128	28, 0.713	17, 0.128	22, 0.318	19, 0.189
**c: Bacilli**	26, 0.564	**11, 0.031**↑	19, 0.189	15, 0.083	16, 0.104	26, 0.564
o: Lactobacillales	**12, 0.041**↑	**2, 0.002**↑	**13, 0.052**↑	**11, 0.031**↑	29, 0.793	30, 0.875
f: Lactobacillaceae	**12, 0.041**↑	**2, 0.002**↑	**13, 0.052**↑	**11, 0.031**↑	29, 0.793	30, 0.875
**g:** ***Ligilactobacillus***	**8, 0.014**↑	**9, 0.018**↑	**0, 0.001**↑	**11, 0.031**↑	20, 0.227	23, 0.372
**g:** ***Lactobacillus***	**13, 0.052**↓	30, 0.875	22, 0.318	28, 0.731	**13, 0.052**↓	24, 0.431
**g:** ***Limosilactobacillus***	**12, 0.041**↓	23, 0.372	26, 0.529	32, 0.958	**11, 0.027**↓	23, 0.372
o: Erysipelotrichales	21, 0.270	31, 0.958	25, 0.462	25, 0.495	26, 0.564	21, 0.270
f: Erysipelotrichaceae	20, 0.227	32, 0.958	25, 0.495	22, 0.318	26, 0.564	21, 0.270
**g:** ***Allobaculum***	15, 0.083	24, 0.431	31, 0.958	25, 0.495	28, 0.713	21, 0.270
**g:** ***Dubosiella***	27, 0.637	29, 0.793	26, 0.564	14, 0.066	27, 0.637	**10, 0.024**↑
**g:** ***Ilebacterium***	24, 0.401	29, 0.753	14, 0.066	23, 0.372	25, 0.495	29, 0.753
**c: Clostridia**	28, 0.713	15, 0.083	20, 0.227	31, 0.958	17, 0.128	26, 0.564
o: Lachnospirales	26, 0.564	15, 0.083	20, 0.227	27, 0.637	20, 0.227	27, 0.637
f: Lachnospiraceae	26, 0.564	15, 0.083	20, 0.227	27, 0.637	20, 0.227	27, 0.637
**g:** ***LachnospiraceaeNK4A***	25, 0.495	17, 0.128	29, 0.793	31, 0.958	21, 0.270	25, 0.495
**g:** ***Roseburia***	29, 0.793	16, 0.093	22, 0.294	23, 0.345	17, 0.115	30, 0.875
**g:** ***A2***	18, 0.141	21, 0.270	22, 0.318	30, 0.834	**11, 0.027**↑	22, 0.294
o: Oscillospirales	25, 0.495	**12, 0.041**↓	16, 0.104	30, 0.875	17, 0.128	22, 0.318
f: Oscillospiraceae	26, 0.564	**9, 0.018**↓	20, 0.227	29, 0.793	15, 0.083	25, 0.495
p: **Actinobacteriota**	31, 0.958	17, 0.128	14, 0.066	16, 0.104	20, 0.227	21, 0.270
**c: Actinobacteria**	14, 0.066	30, 0.875	**4, 0.004**↑	**7, 0.010**↑	24, 0.431	20, 0.227
o: Bifidobacteriales	14, 0.066	31, 0.916	**4, 0.004**↑	**7, 0.010**↑	24, 0.431	20, 0.227
f: Bifidobacteriaceae	14, 0.066	31, 0.916	**4, 0.004**↑	**7, 0.010**↑	24, 0.431	20, 0.227
**g:** ***Bifidobacterium***	14, 0.066	31, 0.916	**4, 0.004**↑	**7, 0.010**↑	24, 0.431	20, 0.227
**c: Coriobacteriia**	20, 0.227	27, 0.637	**6, 0.007**↓	19, 0.189	24, 0.431	**8, 0.014**↓
o: Coriobacteriales	20, 0.227	27, 0.637	**6, 0.007**↓	19, 0.189	24, 0.431	**8, 0.014**↓
f: Eggerthellaceae	25, 0.495	26, 0.564	**8, 0.014**↓	23, 0.372	25, 0.495	16, 0.104
**g:** ***Enterorhabdus***	27, 0.637	19, 0.189	26, 0.564	30, 0.875	27, 0.637	20, 0.227
p: **Proteobacteria**	22, 0.318	20, 0.227	23, 0.372	24, 0.431	16, 0.104	20, 0.227
**c: Gammaproteobacteria**	27, 0.600	18, 0.156	22, 0.318	30, 0.875	16, 0.104	20, 0.227
o: Enterobacterales	15, 0.083	30, 0.875	32, 1.000	24, 0.401	16, 0.104	25, 0.462
f: Enterobacteriaceae	21, 0.248	32, 1.000	32, 1.000	24, 0.401	20, 0.227	25, 0.462
**g:** ***Escherichia-Shigella***	21, 0.248	32, 1.000	26, 0.529	28, 0.713	20, 0.227	22, 0.318

Differences considered statistically significant (*p* ≤ 0.05) are bolded (↑ indicates an increase, ↓ indicates a decrease).

Significant differences, observed in more than one congenic group of the JAX008623 strain compared to C57BL/6J mice, were as follows (Table 1): 1) consistent increase in taxa Lactobacillales/Lactobacillaceae*/Ligilactobacillus* in Tg and NonTg groups, regardless of housing conditions; 2) increased abundance of Actinobacteria/Bifidobacteriales/Bifidobacteriaceae/*Bifidobacterium* in both TgSGH and NonTgSGH groups; 3) decreased abundance of Bacteroidota/Bacteroidia/Bacteroidales, along with an increase in Firmicutes/Bacteroidota ratio (Table 1 and Supplementary Fig. 2B), in both NonTgMGH and NonTgSGH groups; 4) decreased abundance of the members of family Muribaculaceae in both TgMGH and TgSGH groups. Additional changes, observed as specific for one of 4 congenic groups, compared to C57BL/6J group, were: 1) increased abundance of Bacteroidaceae/*Bacteroides* and decreased abundance of genera Lactobacillus and Limosilactobacillus in TgMGH group; 2) lower abundance of Oscillospirales/Oscillospiraceae in the NonTgMGH; 3) lower abundance of class Coriobacteriia followed by ordo Coriobacteriales and Eggerthellaceae in TgSGH group.

Significant differences observed in Tg compared to NonTg group for MGH conditions were (Table 1): increased abundance of Prevotellaceae, Bacteroidaceae/*Bacteroides* and *A2*, along with in lower abundance of genera Lactobacillus and Limosilactobacillus.

Significant differences observed in Tg compared to NonTg group for SGH conditions were (Table 1): increased abundance of *Dubosiella* and decreased abundance Coriobacteriia/Coriobacteriales.

### Differentially abundant taxa at the species classification level

**Fig. 5:**
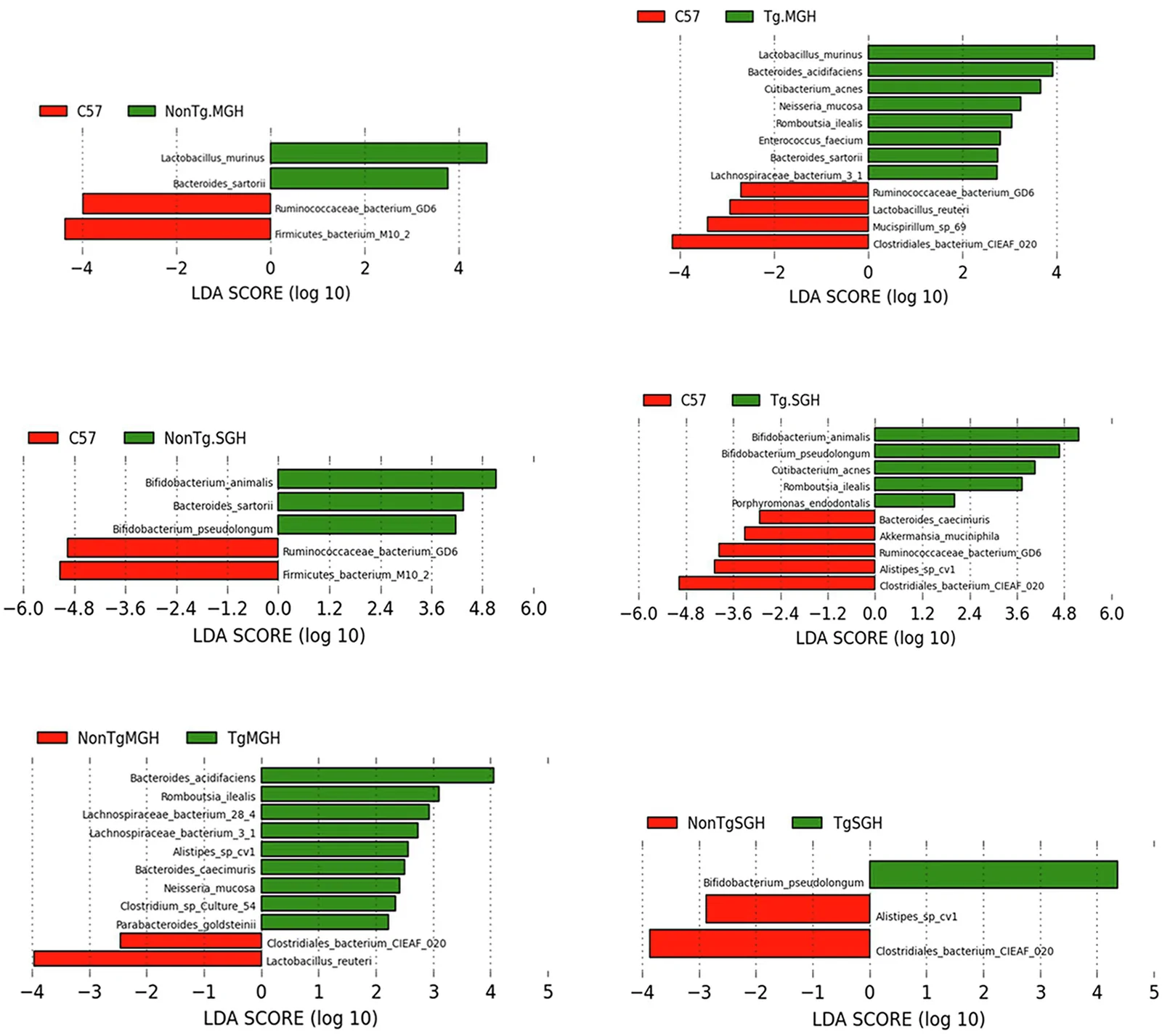
Linear discriminant analysis of effect size (LEfSe) based on OTUs at species level, used to emphasize the differences in fecal microbiota between groups. Tg – transgenic mice, NonTg – non-transgenic mice, MGH – mixed genotype housing; SGH – single-genotype housing. LDA score set at 2.0, *n* = 8 per group.

LEfSe was performed and, based on the LDA score threshold of 2, following species were more abundant in congenic groups of the JAX006823 strain compared to C57BL/6J mice (Fig. 5): in TgMGH group eight species, including *Lactobacillus murinus*, *Bifidobacterium acidifaciens*, *Cutibacterium acnes*, *Neisseria mucosa*, *Romboutsia ilealis*, *Enterococcus faecium*, *Bacteroides sartorii* and *Lachnospiraceae bacterium 3_1*; in NonTgMGH group, *Lactobacillus murinus* and *Bacteroides sartorii*; in TgSGH group five species, including *Bifidobacterium animalis*, *Bifidobacterium pseudolongum*, *Cutibacterium acnes*, *Romboutsia ilealis* and *Porphyromonas endodontalis*; in NonTgSGH group, *Bifidobacterium animalis, Bifidobacterium pseudolongum* and *Bacteroides sartorii*.

In MGH conditions (Fig. 5, third row), significantly higher abundance of *Bacteroides acidifaciens*, *Bacteroides caecimuris*, *Lachnospiraceae bacterium 28_4*, and *Lachnospiraceae bacterium 3_1* was noted in Tg compared to NonTg mice, which made up more than 40% of the prevailing species. In SGH conditions (Fig. 5, third row), only *Bifidobacterium pseudolongum* was significantly increased in Tg compared to NonTg mice.

## Discussion

By introducing biologically relevant variations in the experimental design, this study demonstrated that overexpression of human mutated A53T alpha-synuclein in the transgenic/congenic JAX006823 strain at the premotor stage is associated with robust behavioral symptoms, i.e. coexistence of consummatory anhedonia and sensation-seeking-like behavior, but not with robust gut dysbiosis. It was observed with either Prevotellaceae, Bacteroidaceae/*Bacteroides*, *Lactobacillus*, *Limosilactobacillus* and *A2* as the affected bacterial taxa in one experimental setting or Coriobacteriia/Coriobacteriales and *Dubosiella* in the other and was further confirmed at the species level. These findings indicate that there is no clear link between behavioral phenotype and microbiota in this prodromal PD model, at least at the examined stage. Moreover, characteristics of the JAX006823 strain associated with congenicity were observed both at the behavioral and gut microbiota level, through consummatory hypohedonia and an increase in the relative abundance of Lactobacillales/Lactobacillaceae/*Ligilactobacillus*, demonstrating the importance of non-transgenic littermates for the internal validity of the findings. Through a comprehensive approach, this study identifies challenges in novel strategies seeing the gut microbiota as early diagnostic and therapeutic target for the PD and highlights the potential of the examined transgenic strain in studying low hedonic tone- and sensation-seeking-associated PD prodrome, building the basis for personalized medicine.

Hyperactivity of mutated A53T alpha-synuclein carrier mice has been consistently reported throughout the literature [30, 31]. The current study showed that novelty-induced hyperactivity, an indicative of sensation-seeking as a trait [32], represents a trait of this mouse strain supported by its genetic background, which is clearly evident in SGH conditions. This concept has been initially proposed and examined in our previous study [12]. On the other hand, reduced anxiety-like behavior, which was reflected in the reduction of anxiety index and in the active preference for the open arms of the EPM, emerged as a robust genotype-related characteristics of the strain, being evident in Tg mice in both MGH and SGH conditions. Such behavior typically emerges in the spontaneously-hypertensive rat, a validated animal model of ADHD [43, 44], so findings of the present study strongly support and further corroborate our observation that JAX006823 mice carrying the human mutant A53T alpha-synuclein have ADHD-related behavioral phenotype [12]. Interestingly, the time spent in the center of the EPM, which is a measure related to decision-making and waiting capacity in rodents [45, 46] and is associated with the multidimensional construct of impulsivity [47], emerged as significantly higher in SGH than in MGH mice regardless of the genotype, thus implicating that MGH conditions as such might affect decision-making in co-housed littermates. Decreased anxiety-like behavior is tightly related to sensation-seeking, which is categorized as a psychobiological trait featured by the seeking of varied, novel, complex, and intense sensations and experiences, and the willingness to take risks for the sake of such experience [48]. Less anxious reactivity in sensation-seekers has been related to their tendency to boredom susceptibility and enjoyment of novel, less predictable and intense sensory stimulation and experience, with the thrill and adventure seeking being also related to impulsive behavior [48–50]. Risk-taking is a correlate of sensation-seeking that provides information about preferences for risky forms of arousal but is not a primary motive in behavior [37, 48]. Overall, these facts highlight the value of the JAX006823 strain as a prodromal model of the PD with regard to the symptoms of novelty/sensation-seeking (they are positively associated, [51]) since this personality trait is identified in one of three different prodromal subtypes of PD [27, 28].

The consummatory anhedonia, which was reflected in the absence of preference for 1% sucrose solution over the water in the SPT, emerged as the second robust genotype-related characteristics of the strain, being evident in Tg mice in both MGH and SGH conditions. Anhedonia refers to inability to experience pleasure in response to stimuli usually perceived as rewarding [52] and is differentiated into appetitive/motivational and consummatory component [38]. They are neurobiologically dissociable, with motivational component depending on the mesolimbic dopamine and consummatory component being related to mu-opioid systems [53]. Importantly, motivational deficit, which was measured through motivation to eat (a symptom of apathy assessed in the SPT) and motivation to actively cope with an imposed stressful situation (in the TST), was not observed in A53T carrier mice. Therefore, low hedonic tone may be considered a biomarker that overcomes potential motivational deficits in the early prodromal stage in this A53T alpha-synuclein model of PD. Low hedonic tone can produce and maintain future motivational and cognitive problems, including enhancement-seeking behaviors [54–56] which is in agreement with the above discussed sensation-seeking-like behavior of A53T carriers in the EPM test.

Overall, behavioral findings in the present study showed that anhedonia observed in A53T mice of the JAX006823 strain is associated with strong sensation-seeking, i.e. active preference of open arms of the EPM and low anxiety index. The fact that overexpression of human mutated A53T alpha-synuclein in mice is related to reduced anxiety-like behavior and hyperactivity was already reported [31, 57, 58], but has not been described as comorbid with low hedonic tone, which our study did. Knowing that low hedonic tone has been associated with several psychopathologies, including depressive disorders, substance use, and ADHD [55], further work on consequences of low hedonic tone in this model might help to elucidate psychiatric symptoms and impulse control issues in the prodromal PD [1, 2, 13, 14, 27, 28, 59].

Using a comprehensive 16S microbiome analysis, the other part of the study examined gut/fecal microbiota of mice that were behaviorally characterized. There was no differences in richness and effectiveness (alpha diversity metrics) in 6-months-old JAX006823 littermates. This is generally in concordance with findings that the impact of human alpha-synuclein overexpression on the gut bacterial community is evident in mice older than 6 months [60]. Although there are findings that overexpression of human mutated A53T alpha-synuclein is related to significantly lower alpha diversity in 4-weeks-old mice, the issue of control mouse strain was discussed as the weakness of the study [58]. The adequacy of control groups when working with a genetically modified strain is an issue that should not be underestimated, as will be discussed further.

Considering alpha diversity in PD patients, meta-analysis and clinical data studies reported different findings [5, 61]. Certain authors even suggest that the alpha diversity alone is not a good microbiota marker for PD and other disorders in which a limited number of bacteria are involved in the disease etiology [62]. However, lower Shannon diversity index in Tg and NonTg mice compared to wild-type mice, although generally in the range reported for C57BL/6J strains [63] might point to underlying health issues in the congenic strain.

Beta diversity metrics, which are more sensitive to observe differences as compared with alpha diversity metrics [64], showed that there are both the congenicity-related and genotype-related changes in bacterial community in mice of the JAX006823 strain. The analysis of relative abundance confirmed these differences and gave us a better insight into compositional variability between the examined groups.

Regarding the congenicity-related characteristics of the strain, an increase in the relative abundance of Lactobacillales/Lactobacillaceae/*Ligilactobacillus*, lactic acid producing bacteria, emerged as a signature of the JAX006823 strain, independent of the genotype and social grouping. Additionally, the increased abundance of Actinobacteria/Bifidobacteriales/Bifidobacteriaceae/*Bifidobacterium* emerged as an additional, SGH-dependent characteristic of the JAX006823 strain, while a decrease of Bacteroidota/Bacteroidia/Bateroidales taxa, along with a higher Firmicutes/Bacteroidota ratio, emerged as a general signature of non-transgenicity within the JAX006823 strain. Observed findings are fully in agreement with the view that the composition of the gut microbiota behaves as a complex polygenic trait shaped by environmental and host genetic factors [65, 66], with the host genetic background as a fundamental component in determining the composition and profile of the gut microbiota [65]. Our study clearly demonstrated that the transgenic/congenic strain of interest tends to have its own microbiota imprint and, within it, peculiarities in microbiota abundance related to genotype and genotype-based social microenvironment. This was additionally evident from LEfSe analysis, at the species level, with lower abundance of *Ruminococcaceae bacterium GD6* in all groups of Tg and NonTg mice compared to C57BL/6J mice, the enrichment of *Lactobacillus murinus* and *Bacteroides sartorii* as MGH-related and the enrichment of *Bifidobacterium animalis* and *Bifidobacterium pseudolongum* as SGH-related characteristics. Interestingly, increased abundance of *Bifidobacterium* has been found in ADHD [67], which stands good with hyperactive behavior of SGH mice of both genotypes in the OFT. Bifidobacterium have also been linked to improved mood health [68] but the same cannot be said for mice in this study. Overall, the data presented clearly indicate that using C57BL/6J mice as controls, which is generally recommended (https://www.jax.org/strain/006823 last accessed on 21 July 2026), would lead to misinterpretation that α-synuclein overexpression in Tg mice is related to the increased amounts of *Ligilactobacillus* genus, which was introduced in 2020, as part of a major taxonomic reclassification of the *Lactobacillus* genus [69], a potential microbiota signature of the PD (discussed below).

In humans, enrichment of the genera *Lactobacillus*, *Akkermansia*, and *Bifidobacterium* and depletion of bacteria belonging to the Lachnospiraceae family and the *Faecalibacterium* genus, both important short-chain fatty acids producers, emerged as the most consistent PD gut microbiome alterations [5]. The present study did not find robust alterations in the PD-associated microbiome in 6-month-old behaviorally examined JAX006823 mice, although each of two housing conditions resulted in changed gut microbiota in the way described in certain psychiatric conditions associated with prodromal PD. In MGH, we observed increased abundance in families Prevotellaceae (mucin-degrading bacteria shown to be enriched in patients with depression and anxiety [70] and with inconsistent findings in PD [5]) and Bacteroidaceae/*Bacteroides* genus (bacteria with high abundance in PD [71] and ADHD [72]), along with decreased abundance in *Limosilactobacillus* genus (might be related to depression [73]) and *Lactobacillus* genus (reported in ADHD [74]). In SGH, we observed increased abundance in *Dubosiella* genus (key bacteria in gut-originated PD model [75]) along with decreased abundance in Coriobacteriia class, Coriobacteriales ordo (annotated as involved in depression [76] and anhedonia [77]). Compared to NonTg mice, *Bacteroides* and *Lachnospiraceae* species (*28-4* and *3-1*), along with *Romboutsia ilealis* and *Neisseria mucosa*, were favored in TgMGH mice; the abundance of *Lachnospiraceae bacterium 28-4* has recently been positively related to metabolic health [78]. Enrichment of *Bifidobacterium pseudolongum* appeared as a hallmark of the microbiota of TgSGH mice. This finding is particularly important considering that enrichment of *Bifidobacterium* species in PD has been related to levodopa treatment, rather than to a primary mechanism of the disease development (reviewed in [26]). Our findings implicate that increased abundance of *Bifidobacterium pseudolongum* is related to the disease prodrome, but it is not obligatory. Moreover, *Bifidobacterium pseudolongum* is considered a potential therapeutic strategy for colorectal cancer [79, 80] and further work in the A53T mouse model may help to understand the lower risk of developing colorectal cancer in some PD patients [81]. The obtained data suggest that enrichment of health-promoting bacteria is part of the prodrome-associated dysbiosis, perhaps as a natural defense mechanism; the duration and extent of these changes within the disrupted microbiota balance may be additional parameters to consider.

Overall, presented results imply that there is no clear link between the behavioral phenotype, characterized by coexistence of low hedonic tone and sensation-seeking, and the gut microbiota in this prodromal model of PD, at least in the phase examined. Although there are implications that overexpression of wild-type human alpha-synuclein can drive an age-dependent dysbiosis in mice [60], there are also findings that the gut microbiota related with overexpression of A53T mutant human alpha-synuclein has a very limited effect on behavioral phenotype of the host and our study supports this observation [58]. This leaves the possibility that complex behavioral disturbances related to A53T alpha-synuclein overexpression in the early prodrome are accompanied by, but not dependent on, gut dysbiosis. The fact that A53T mice have increased food/calorie intake compared to NonTg mice, as observed in our study, is already known and has been explained by increased energy expenditure [82]; however, this appears to be a context-dependent feature, as it was not evident under SGH conditions. Furthermore, weight maintenance in NonTg mice may be aided by increased Firmicutes/Bacteroidota ratio, an indicator of a greater capacity to harvest energy from food [83], which certainly deserves further analysis.

This study, while providing relevant insights for the field, has limitations. First, only male mice were used in the experiment, which limits external validity of the observed findings. Second, only one age group of male mice was examined, which prevents insight into the changes that follow. However, despite these limitations, the results obtained provide important key takeaways indicating that: 1) genotype-based social grouping may lead to different functional presentations in an experiment with prolonged duration, given that each condition has its own microbiota autograph; 2) the selection of the control group has a constitutive role in the internal validity of the study and should therefore receive more attention in order to serve the purpose and contribute to the generation of reliable, unbiased and objective results.

In conclusion, this deliberately designed study yielded results implicating that the overexpression of a pathological form of alpha-synuclein, related to the pathogenesis of the PD, is associated with a robust premotor behavioral phenotype expressed as co-occurrence of consummatory anhedonia and sensation-seeking/risk-taking behavior, without significant motivational deficits and with variable gut dysbiosis, which identifies challenges in novel strategies seeing the gut microbiota as early diagnostic and therapeutic target for the PD. Nevertheless, the examined transgenic strain has a potential in studying low hedonic tone- and sensation-seeking-related behavior associated with the PD prodrome, providing an experimental platform to test tailored therapies and building the basis for personalized medicine. Hopefully, this study will help raise awareness of the importance of methodological approaches in improving the validity and translatability of preclinical research.

## Supplementary information

Supplementary material

## Data Availability

The data for this study have been deposited in the European Nucleotide Archive (ENA) at EMBL-EBI under accession number PRJEB104318 (https://www.ebi.ac.uk/ena/browser/view/PRJEB104318), and secondary accession number ERP185613. Other data that support the findings of this study are available from the corresponding author upon reasonable request.
