# Supplementary material for: Coexistence of anhedonia and sensation-seeking with context-dependent gut dysbiosis in a prodromal transgenic mouse model of Parkinson’s disease
Source: Transl Psychiatry. 2026 Jul 27;16:580. doi: 10.1038/s41398-026-04306-w (PMC13687092; doi:10.1038/s41398-026-04306-w)
Supplement: Supplementary file 1 [file 41398_2026_4306_MOESM1_ESM.docx]

**Supplementary information**

**Supplementary Figure 1. Graphical presentation of the experimental design.** Experimental day 1: fecal samples were collected from mice that, after being placed in a clean cage, provided at least 2 fecal boli. Experimental day 2: mice were individually placed in the open field cage and allowed to freely explore for 30 minutes; the data were automatically recorded (Auto-Track System, Columbus Instruments, Columbus, OH, USA). Experimental day 3: mice were individually placed in the elevated plus maze and allowed to freely explore for 10 minutes; video-recording was made and films were subsequently analyzed. Experimental day 4: mice were fixed by the tail and their behavior was recorded for 5 minutes; the recorded material was subsequently analyzed. Experimental day 5: the sucrose preference test was performed on mice that were individually housed for 48 hours, during which they were allowed 2-bottle-choice (water and 1% sucrose solution); consumption was measured at 24 hours, when the position of the bottles in the feeder was rotated; the average of two measures was used as a final result.

**Supplementary Figure 2.** Composition of fecal microbiota of transgenic and non-transgenic mice of the JAX006823 strain and C57BL/6J mice. (A) Phylum level; (B) Firmicutes/Bacteroidota ratio,

*p=0.041, **p=0.005, U-test; (C) Ordo level; (D) Family level; (E) Genus level; n = 8 per group.

Tg – transgenic, NonTg – non-transgenic, MGH – mixed-genotype housing, SGH – single-genotype housing.

**Supplementary Figure 3. Total calorie intake (kcal) per body weight (g) in transgenic and non-transgenic mice of the JAX006823 strain and C57BL/6J mice during the sucrose preference test.** Results are presented as mean ± SD. Throughout the duration of the experiment, mice were fed pelleted mouse chow formulated with at least 20% protein and at least 5% fat (more details are provided in the general methodological information, section below). Tg – transgenic, NonTg – non-transgenic, MGH – mixed-genotype housing, SGH – single-genotype housing. n = 12 per group; ^#^p<0.05, Tukey HSD test; ^c^p<0.05 *vs*. TgSGH, *t*-test.

**General methodological information**

Depending on the group assignment, male mice of different genotypes (Tg, NonTg) were either co-housed within a cage (mixed-genotype housing (MGH), balanced by genotype) or separated by genotype (single-genotype housing, SGH). Groups were formed with P29±1 day old mice considering that, in addition to the first weeks of life, a period between weaning and young adulthood is another window of opportunity to influence microbiome composition [29]. A home-cage group composition (4-6 mice per cage) was not changed until the end of the experiment. C57BL/6J mice were housed under the same conditions and were not co-housed with mice of the JAX006823 strain. Throughout the duration of the experiment (and as standard in the institutional vivarium), mice were fed pelleted mouse chow (produced in the GEBI DOO PO ČANTAVIR animal feed factory) formulated with min 20% protein diet, min 5% fat, max 13.5 moisture, max 5% crude fiber and max 8% ash (detailed composition is available upon request; metabolizable energy: 3218 kcal/kg).

The number of animals per group for behavioral analysis (12 mice per experimental group) and microbiota analysis (8 mice per experimental group) was defined to be large enough to draw conclusions while being mindful of resources/animals, taking into account our previous experience with the strain [12, 24] and guidelines for optimizing experimental design for microbiota analysis [29]. No statistical methods were used to estimate sample size.

Being aware that both cage and litter effects may influence the experimental findings, the offspring from different litters (at least two, randomly selected and matched by date of birth) were cage mates and several cages were used to build experimental groups. For single genotype housing (SGH, 12 mice per group/genotype), 3 cages with 4 animals (of a given genotype) per cage formed a group; for microbiome analysis, no more than 3 mice per cage, randomly selected, were used to achieve the number of 8 mice per group/genotype (3+3+2). For mixed genotype housing (MGH, 12 mice per group/genotype), 5 cages with 4-6 animals per cage, balanced by genotype, formed MGH groups; for microbiome analysis, 3 mice per cage, randomly selected, were used (2Tg + 1NonTg or 1Tg + 2NonTg) except in one cage where the entire cage was used (2Tg + 2NonTg).

All behavioral tests, except the sucrose preference test, were conducted in the testing room. Before the start of the procedures, mice were allowed to habituate (in their home cages) to the conditions of the testing room during the period for 30 minutes. The sucrose preference test was conducted in an experimental room within the animal facility. Fecal sample collection and all behavioral testing, except sucrose preference test, took place between 9:00 and 14:00.

The experiments, in the described scope, were conducted once, that is, they were not replicated in the laboratory.

References:

12. Dubljević O, Pavković Ž, Srbovan M, Potrebić M, Stanojlović M, Pešić V. Attention-deficit/hyperactivity disorder-related psychomotor activity and altered neuronal activity in the medial prefrontal cortex and striatum in the A53T mouse model of Parkinson's disease and other synucleinopathies: Findings from an "endophenotype" approach. Prog Neuropsychopharmacol Biol Psychiatry <https://doi.org/10.1016/j.pnpbp.2025.111273> (2025).

24. Dubljević O, Dragoj M, Potrebić Stefanović M, Srbovan M, Stanojlović M, Pavković Ž. Genotype-Based Housing as a Potential Confounder in Studies Using Transgenic Mouse Models-Insight from the A53T Mouse Model of Parkinson's Disease. Biomedicines <https://doi.org/10.3390/biomedicines13061506> (2025).

29. Laukens D, Brinkman BM, Raes J, De Vos M, Vandenabeele P. Heterogeneity of the gut microbiome in mice: guidelines for optimizing experimental design. FEMS Microbiol Rev 2016; 40: 117-132.

**Exclusion criteria (pre-established)**

Humane endpoints – Termination criteria: animal health was monitored on a daily basis during the experiment using a screening Questionnaire for Monitoring Stress in Laboratory Animals available in [24]. The animals would have been excluded from the experiment and euthanized if it was exhibiting scores greater than 5. None of the mice reached our predefined termination criteria.

Exclusion criteria based on technical difficulties: any animals whose recordings were compromised or unusable for the analysis; a spillage of water/sucrose solution in the cage.

*One animal from the NonTgSGH group was excluded from the analysis of EPM data due to a corrupted video recording of behavior in the EPM test. None of the mice were excluded based on the second criterion.

Exclusion criteria based on absence of data: animal that did not show any activity in the OFT, EPM or TST tests; animal that did not show any consummatory activity towards water, sucrose solution and/or food.

*None of the mice were excluded based on this criterion.

References:

24. Dubljević O, Dragoj M, Potrebić Stefanović M, Srbovan M, Stanojlović M, Pavković Ž. Genotype-Based Housing as a Potential Confounder in Studies Using Transgenic Mouse Models-Insight from the A53T Mouse Model of Parkinson's Disease. Biomedicines <https://doi.org/10.3390/biomedicines13061506> (2025).

**Fecal sample collection**

Fecal samples were collected from mice that, after being placed in a clean cage, provided at least 2 fecal boluses which were then stored in empty sterile 1.5 ml Eppendorf tubes at -80°C until further use. For this analysis, the number of mice per group is 8 and they were randomly selected (please see the "General methodological information" section provided above).

**Open field test (OFT)**

Following a habituation period to the testing room, mice were placed in Opto-Varimex cages (44.4 × 43.2 × 20 cm, Columbus Instruments, Columbus, OH, USA) and allowed to freely explore the novel environment for 30 minutes, representing a habituation phase (Mejias et al., 2021; Dubljević et al., 2025). Each cage was equipped with 15 infrared emitters located on the X and Y axes, with an equivalent number of receivers located on the opposite walls of the cage to track the full horizontal activity in a single cage. The additional Z axis sensors, placed above the animal, measured the vertical activity. The data were recorded and analyzed using the Auto-Track software (ATM3, version 4.51, Columbus Instruments).

Parameters of interest in the OFT were: locomotor activity, vertical activity. The adequacy of these parameters in examining exploratory activity in rodents in response to a novel environment was extensively discussed in previous publications by our group (Potrebić et al. 2022; Dubljević et al., 2025).

After the termination of testing period mice were returned to their home cages in agreement with previously defined genotype-related housing conditions. The equipment was cleaned to eliminate any scent traces from previously used animals, to be ready for the next testing.

References:

Mejias R, Rodriguez-Gotor JJ, Niwa M, Krasnova IN, Adamczyk A, Han M et al. Increased novelty-induced locomotion, sensitivity to amphetamine, and extracellular dopamine in striatum of Zdhhc15-deficient mice. *Transl Psychiatry* <https://doi.org/10.1038/s41398-020-01194-6> (2021).

Dubljević O, Pavković Ž, Srbovan M, Potrebić M, Stanojlović M, Pešić V. Attention-deficit/hyperactivity disorder-related psychomotor activity and altered neuronal activity in the medial prefrontal cortex and striatum in the A53T mouse model of Parkinson's disease and other synucleinopathies: Findings from an "endophenotype" approach. *Prog Neuropsychopharmacol Biol Psychiatry* <https://doi.org/10.1016/j.pnpbp.2025.111273> (2025).

Potrebić MS, Pavković ŽZ, Srbovan MM, Dmura GM, Pešić VT. Changes in the Behavior and Body Weight of Mature, Adult Male Wistar Han Rats after Reduced Social Grouping and Social Isolation. *J Am Assoc Lab Anim Sci* 2022; 61: 615-623.

**Elevated plus maze test (EPM)**

The EPM apparatus consisted of two opposite open arms (35 x 5 cm) and two opposite closed arms (35 x 5 x 15 cm) connected by a central platform (5 x 5 cm). The apparatus is made out of light gray plexiglass and is elevated 50 cm above the floor.

Testing was conducted during the light phase of the cycle. Each mouse was placed in the central area of the maze facing one of the closed arms, then it was allowed to freely explore the maze for 10 min. At the end of each trial, the maze was cleaned with an ethanol solution and wiped clean. Behavior of the animals in the EPM was recorded using a light-sensitive camera (Logitech C270 HD- 960-001063) mounted 1.5 m above the EPM. All recordings were stored on a hard disk until further behavioral analysis, using ANY-maze video tracking software (version 7.2, Stoelting Co., Wood Dale, IL, USA), by a researcher blinded to the experimental groups. To score entries and exits in the open and closed arms, at least 80% of the animal must be in the arm for an entry to occur and at least 70% of the animal must remain in the arm for an exit not to occur.

Behavioral parameters of interest were: number of entries into open and closed arms, distance traveled in open and closed arms, time spent in open and closed arms and in the central area.

A locomotion independent preference for open arms was calculated as: number of open arm entries / (number of open + closed arm entries). Open arms of the EPM are the source of the anxiogenic stimulus (Treit et al., 1993) and are usually avoided (Walf and Frye, 2007). Thus, a preference for open versus closed arms implicates increased motivation and/or weakened inhibition of the animal towards an anxiogenic stimulus, i.e. sensation-seeking/risk-taking behavior (Yen et al., 2013). Risk-taking is a correlate of sensation seeking but is not a primary motive in behavior (Zuckerman, 1994).

The anxiety index was calculated according to Cohen et al. (2013) as follows: Anxiety Index = 1 – [([Open arm time/Test duration] + [Open arms entries/Total number of entries])/2]. Higher activity in the open arms is an indicator of lower anxiety-like behavior.

References:

Treit D, Menard J, Royan C. Anxiogenic stimuli in the elevated plus-maze. *Pharmacol Biochem Behav* 1993; 44: 463-469.

Walf AA, Frye CA. The use of the elevated plus maze as an assay of anxiety-related behavior in rodents. *Nat Protoc* 2007; 2: 322-328.

Yen Y-C, Anderzhanova E, Bunck M, Schuller J, Landgraf R, Wotjak CT. Co-segregation of hyperactivity, active coping styles, and cognitive dysfunction in mice selectively bred for low levels of anxiety. *Front Behav Neurosci* <https://doi.org/10.3389/fnbeh.2013.00103> (2013).

Zuckerman M. *Behavioral expressions and biosocial bases of sensation seeking*. Cambridge university press, 1994.

Cohen H, Matar MA, Joseph Z. *Animal models of post-traumatic stress disorder*. In Current Protocols in Neuroscience, 2013, John Wiley & Sons; chapter 9, unit 9.45. doi: 10.1002/0471142301.ns0945s64.

**Tail suspension test**

Each mouse was suspended for 5 minutes, at a height of 15 cm, by tape attached to the end of its tail. Their behavior was recorded with a light-sensitive camera, and the videos were subsequently manually analyzed by a researcher blinded to the group assignments.

Mobility was defined as movements of the hind legs, and/or other escape oriented behaviors. Immobility was defined as passive hanging without attempts to right itself. Since mice of the C57BL/6J strain may climb their tails during the test, a clear plastic cylinder (4 cm length, 1.5 cm diameter) was placed around their tails to prevent such behavior (Dao et al., 2010).

The latency for the first immobility and total immobility duration were measured. An increased immobility duration or a reduced latency to first immobility are indicative of a depression-like phenotype.

References:

Dao DT, Mahon PB, Cai X, Kovacsics CE, Blackwell RA, Arad M et al. Mood disorder susceptibility gene CACNA1C modifies mood-related behaviors in mice and interacts with sex to influence behavior in mice and diagnosis in humans. *Biol Psychiatry* 2010; 68: 801-810.

**Sucrose preference test**

Sucrose consumption and preference for palatable solutions displays “liking” (consummatory) capacity of hedonic response (Der-Avakian and Markou, 2012; Willner, 2017). Along with sucrose consumption measurements and sucrose preference calculation, we introduced measurement of food consumption (Pavković et al., 2020, Potrebić et al., 2021) considering that 1) food intake is a goal-directed behavior indispensable for life support and reduced feeding can be used as apathy-like symptom (Palmiter, 2008; Yamada et al., 2018), and 2) animals that are not motivated to engage in goal-directed behaviors still have a preference for sucrose (Palmiter, 2008).

The sucrose preference test was performed on mice that were individually housed for 48 hours, during which they were allowed 2-bottle-choice, one containing water and the other a 1% sucrose solution. Consumption was measured at 24 hours, when the position of the bottles in the feeder was rotated. Animals were not deprived of food or water before testing to avoid any stress related to these procedures.

The liquids consumed per cage/animal were calculated by subtracting the volume left in the bottle from the initial volume (100 ml).

The food consumed was calculated by subtracting the amount of food that was left from the amount of food initially placed (50 g). When placing food in the feeder, whole pellets were selected to avoid small pieces of pellets falling through the feeder grate, but at each measurement, the cage was checked for pieces of food and, if there were any, the pieces found were measured as uneaten.

The following parameters were calculated for each 24-hour period:

- food consumption (g) per BW (g)
- volume of sucrose consumed / (volume of sucrose consumed + volume of water consumed), with obtained preference values ranging from 0.5 (no preference) to 1 (complete preference).

The mean of the two measures/preferences was used as the final result.

References:

Der-Avakian A, Markou A. The neurobiology of anhedonia and other reward-related deficits. Trends Neurosci 2012; 35: 68-77.

Willner P. Reliability of the chronic mild stress model of depression: A user survey. *Neurobiol Stress* 2016; 6: 68-77.

Pavković Ž, Potrebić M, Kanazir S, Pešić V. Motivation, risk-taking and sensation seeking behavior in propofol anesthesia exposed peripubertal rats. *Prog Neuropsychopharmacol Biol Psychiatry 2020; 96: 109733.*

Potrebić M, Pavković Ž, Lončarević-Vasiljković N, Kanazir S, Pešić V. Altered hedonic, novelty-, stress- and D-amphetamine-induced response due to social isolation in peripuberty. *Prog Neuropsychopharmacol Biol Psychiatry* 2021; 108: 110186.

Palmiter RD. Dopamine signaling in the dorsal striatum is essential for motivated behaviors: lessons from dopamine-deficient mice. Ann N Y Acad Sci 2008; 1129: 35-46.

Yamada C, Mogami S, Kanno H, Hattori T. Peptide YY Causes Apathy-Like Behavior via the Dopamine D2 Receptor in Repeated Water-Immersed Mice. Mol Neurobiol 2018; 55: 7555-7566. <https://doi.org/10.1007/s12035-018-0931-1>.

**Bacterial DNA extraction and sequencing**

Frozen fecal samples were used for metagenomic DNA extraction with ZR Fecal/Soil Microbe MiniPrep™ Kit (Zymo Research Corp., Irvine, CA USA), according to the manufacturer’s guidelines. Samples were then diluted to recommended concentration of 12 ng/μl, and 20 μl of final volume was sent to Novogene Company (Cambridge, United Kingdom). V3-V4 hypervariable regions of 16S rRNA gene were amplified using 341F (forward, 5’–CCTAYGGGRBGCASCAG–3’) and 806R (reverse, 5’–GGACTACNNGGGTATCTAAT– 3’) specific primers with the barcode. Sequencing libraries were prepared and paired-end sequencing was performed on Illumina NovaSeq 6000 platform. Paired-end reads were merged using FLASH (V1.2.11, http://ccb.jhu.edu/software/FLASH/) (Magoč and Salzberg, 2011). High-quality Clean Tags were obtain with the fastp (Version 0.23.1) software (Bokulich et al., 2012). For the taxonomic classification SILVA Database (https://www.arb-silva.de/) was used. OTUs abundance data were normalized before the alpha and beta diversity analysis. The top 10 taxa at Phylum, Order and Family level and top 30 taxa at Genus level were selected to plot relative abundance distribution histogram in Perl through SVG function. Venn and Flower diagrams were done in R with Venn Diagram function and in perl with SVG function, respectively. Alpha diversity indices Observed-species, Chao1 and Shannon, and Beta diversity were calculated by QIIME (Version 1.9.1) (Caporaso et al., 2010) and displayed with GraphPad Prism 6 and R software (Version 4.0.3) correspondingly. Non-parmetric Adonis test was performed using R vegan and ggplot2 package. Linear discriminant analysis effect size (LEfSe), was performed using the Galaxy platform (The Huttenhower Lab, 2021).

References:

Magoč T, Salzberg SL. FLASH: fast length adjustment of short reads to improve genome assemblies. *Bioinformatics* 2011; 27: 2957-2963.

Bokulich NA, Subramanian S, Faith JJ, et al. Quality-filtering vastly improves diversity estimates from Illumina amplicon sequencing. *Nat Methods* 2013; 10: 57-59.

R Core Team (2021) R: A Language and Environment for Statistical Computing. R Foundation for Statistical Computing, Vienna. https://www.R-project.org/

Caporaso JG, Kuczynski J, Stombaugh J, Bittinger K, Bushman FD, Costello EK et al. QIIME allows analysis of high-throughput community sequencing data. *Nat Methods* 2010; 7: 335-336.

LEfSe platform from Galaxy. Harvard.: The Huttenhower Lab; 2021. https://huttenhower.sph.harvard.edu/galaxy/
